# Rootstock selection shapes melon taste by divergent regulation of sugar and amino acid metabolism

**DOI:** 10.3389/fpls.2026.1740115

**Published:** 2026-05-01

**Authors:** Hao Zhang, Kaleem Muhammad Mohsin, Qigan Liang, Yinke Du, Paerhati Maerhaba, Banlv Tian, Haojie Zhang, Wenwei Zhang, Yuan Huang, Min Wang, Jingrong Zhu, Xiaofa Fu

**Affiliations:** 1 Key Laboratory of Functional Nutrition and Health of Characteristic Agricultural Products in Desert Oasis Ecological Region (Co-construction by Ministry and Province), Ministry of Agriculture and Rural Affairs Xinjiang China, Urumqi, China; 2 Breeding Station in Sanya, Hainan, Xinjiang Academy of Agricultural Sciences, Xinjiang Uygur Autonomous Region, Sanya, China; 3 Sanya Mingzhu Melon and Watermelon Variety Demonstration Evaluation and Research Center, Sanya, China; 4 National Key Lab for Germplasm Innovation and Utilization of Horticultural Crops, College of Horticulture and Forestry Sciences, Huazhong Agricultural University, Wuhan, Hubei, China; 5 Vegetable Research Institute, Hainan Academy of Agricultural Sciences, Haikou, China; 6 Key Laboratory of Genetic Resources Evaluation and Utilization of Tropical Fruits and Vegetables (Co-construction by Ministry and Province), Ministry of Agriculture and Rural Affairs, Haikou, China

**Keywords:** *Cucumis metuliferus*, grafting, metabolomics, rootstock, transcriptomics

## Abstract

**Background:**

Melon (*Cucumis melo L*.) is a widely cultivated fruit globally, valued for its high nutritional content and diverse culinary uses. However, the molecular mechanisms underlying flavor enhancement mediated by grafting remain poorly understood.

**Objective:**

This study aimed to elucidate the molecular regulatory mechanisms of flavor formation in melons grafted onto different rootstocks: *Cucurbita moschata* (QY1) and *Cucumis metuliferus* (ZM4).

**Methods:**

Transcriptomic and metabolomic analyses were integrated to systematically dissect dynamic changes in gene expression and metabolite accumulation in grafted systems (ZM4/SG, QY1/SG).

**Results:**

The two rootstocks induced distinct genetic and metabolic adaptation strategies. The ZM4 rootstock activated genes related to carbohydrate metabolism, such as sucrose-cleaving enzymes (*INV, bglX/bglB*) and nucleotide-sugar synthases (*UGDH, GAE*), promoting the accumulation of glucose and fructose and expanding UDP-sugar precursors for glycosylation. These genetic changes enhanced anabolic flux, leading to the accumulation of cell wall polysaccharides (e.g., *mannan*) and high-value *glycosides* (e.g., *vanilloyl* glucose). Additionally, ZM4/SG exhibited enhanced stress resilience via activation of AKR1A1 and *MIOX*, resulting in increased accumulation of xylitol and trehalose-6-phosphate. In contrast, the QY1 rootstock activated energy metabolism pathways, upregulating E3.2.1.21 and *MIOX* to promote glycoside hydrolysis and NADPH regeneration, thereby strengthening energy metabolism.

**Discussion:**

This study clarifies the mechanism of rootstock-mediated metabolic flux reprogramming: ZM4/SG coordinates hydrolytic, biosynthetic, and stress-responsive pathways to redirect carbon flux toward structural polysaccharides and high-value glycosides, providing molecular targets for improving melon aroma and flavor quality. The results align with the hypothesis that rootstocks regulate fruit quality traits and establish a working model for understanding synergistic regulatory networks between rootstocks and scions. This lays a theoretical foundation for developing precise quality modulation strategies in melons.

## 1 Introduction

*Cucumis melo* L., commonly known as melon, holds significant global stature as a key horticultural crop within the Cucurbitaceae family. It is renowned for its culinary versatility and rich nutritional profile, boasting an impressive annual production nearing 30 million tons (Zhang et al., 2019). Attributes showcased by melon fruits encompass size, shape, color, sugar and acid contents, and accumulation of diverse aroma volatiles (Ma et al., 2022). To meet the demands of local and international markets, ongoing improvements in production techniques are necessary to provide high-quality fruit (Gao et al., 2019). Producing high-quality fruits requires in-depth research into the processes and mechanisms behind fruit quality development, as well as advances in techniques to improve flavor, nutritional value, and overall quality.

Grafting is a cornerstone technique in Solanaceous and Cucurbitaceous vegetable production, which unites a scion and a rootstock to combine their advantageous traits and cultivate plants with enhanced vigor and agronomic performance (Bie et al., 2025; Bie ZhiLong et al., 2017). It is primarily employed to enhance plant vigor and yield, most notably by conferring resistance to soil-borne diseases and other biotic and abiotic stresses (Shireen et al., 2020; Nawaz et al., 2017). Under low-temperature stress, the *Ketol-acid reductoisomerase 1*(*CmoKARI1*) protein from pumpkin rootstock can be transported to cucumber scions, enhancing their cold tolerance (Zhang et al., 2025); meanwhile, *calcium-dependent protein kinases* (*CmoCDPK20*) regulates the expression of genes related to jasmonic acid synthesis and antioxidant enzymes by phosphorylating the transcription factor *dehydration-responsive element-binding protein 2A* (*CmoDREB2A*), thereby improving the salt tolerance of grafted cucumbers (Chen et al., 2025). In tomatoes, grafting onto MSH-1 rootstock increased progeny yield by 35%, and this yield-enhancing effect remained stable up to the fifth generation (Kundariya et al., 2020).

Grafting also profoundly influences a multitude of quality attributes, encompassing taste, aroma, and nutritional content (Guo et al., 2024). Sugar and acid are considered the basic compounds for fruit sweetness (Tikunov et al., 2020). The sweet taste is primarily determined by sugars, including glucose, sucrose, and fructose (Cheng et al., 2020). Glutamic acid and aspartic acid are the most abundant amino acids in the melon protein concentrates (Wan et al., 2022). Research delving into the metabolic profiles of grafted melons reveals that specific rootstock-scion combinations can significantly elevate levels of primary metabolites, such as sugars and organic acids, crucial determinants of the fruit’s overall quality (Kaleem et al., 2022; Aslam et al., 2020; Camalle et al., 2023). For instance, using yellow-seeded pumpkin as a rootstock significantly improves the fruit quality of cucumber (Liu et al., 2025). Pumpkin rootstock also helps improve the quality of grafted watermelon (Aslam et al., 2020; Zhong et al., 2019). Melon grafted onto pumpkin rootstocks tends to exhibit a reduced odor intensity and lower consumer preference scores when compared to those from self-grafted (SG) plants. While certain aromatic volatile compounds that contribute to a sweet aroma may be less prevalent in pumpkin-grafted melons, undesirable compounds with unpleasant odor characteristics may be increased (Ogunbusola et al., 2022).

The exploration of alternative rootstocks for *Cucurbita* hybrids should prioritize resistance to soil-borne pathogens and the enhancement of fruit quality related traits. This study employs a multi-omics strategy to elucidate the mechanisms by which distinct rootstocks—the low-quality associated QY1 and the high-quality associated ZM4 regulate fruit quality related traits (Wang et al., 2025). We aim to delineate how they differentially modulate the accumulation of key taste-related metabolites, specifically sugars and amino acids, and expression of genes in their biosynthetic pathways.

## 2 Materials and methods

The experiment was conducted in a plastic greenhouse at the Hainan Sanya Breeding Station of the Xinjiang Academy of Agricultural Sciences, located at 18°14’3” N, 109°30’2” E. A thick-skinned melon cultivar, ‘DuMi’(DM) (*Cucumis melo* L.) from Xinjiang Mingxin Kehong Agricultural Technology Co., Ltd, was used as the scion. The rootstock seeds were sown in plastic plug trays five days before planting the scion seeds. Grafting was performed using the hole insertion method upon the emergence of the first true leaf (Kaleem et al., 2023). The randomized complete block design (RCBD) with three blocks (50 plants per block) was used in this experiment. Each grafting combination was considered as a treatment. In this study, self-grafted seedlings of the melon cultivar ‘DM’ were used as the control. A total of three fruits were harvested from each replication (nine fruits per treatment). At harvest time, the blossom end of mature fruits turned soft, revealing a fully developed abscission zone marked by a crack around the peduncle. This aligns with the standard melon management procedure. After harvesting, the fruits were transported in refrigerated conditions to Huazhong Agricultural University, Wuhan, China, for further physiological and biochemical analyses. The juice of fruits was extracted by squeezer and placed in a beaker. The extracted juice was centrifuged and the supernatant was taken to measure total soluble solid content by putting a drop of supernatant on refractometer (Pocket PAL-1, Atago, Japan).

### 2.1 Non-targeted metabolomic analysis of melon fruit

#### 2.1.1 Metabolite extraction

A solid sample (50 mg) was placed in a 2 mL centrifuge tube along with a 6 mm diameter grinding bead. For metabolite extraction, 400 μL of an extraction solution consisting of methanol and water (4:1, v/v) and containing 0.02 mg/mL of the internal standard (L-2-chlorophenylalanine) was added. The samples were homogenized using a frozen tissue grinder at -10°C and 50 Hz for 6 minutes. Subsequently, low-temperature ultrasonic extraction was performed at 5°C and 40 kHz for 30 minutes. The mixture was then incubated at -20°C for 30 minutes before being centrifuged at 13,000 g for 15 minutes at 4°C. Finally, the supernatant was collected and transferred to an injection vial for LC-MS/MS analysis.

#### 2.1.2 UHPLC-MS/MS analysis

The LC-MS/MS analysis was performed using a Thermo UHPLC-Q Exactive HF-X system equipped with an ACQUITY HSS T3 column at Majorbio Bio-Pharm Technology Co., Ltd. (Shanghai, China). The mobile phase consisted of solvent A (0.1% formic acid in water:acetonitrile, 95:5, v/v) and solvent B (0.1% formic acid in acetonitrile:isopropanol:water, 47.5:47.5:5, v/v). The analysis was conducted at a flow rate of 0.40 mL/min, with the column maintained at 40°C and an injection volume of 3 μL.

Mass spectrometric data were acquired using a Thermo UHPLC-Q Exactive HF-X mass spectrometer equipped with an electrospray ionization (ESI) source operating in both positive and negative ionization modes. The optimized parameters were as follows: auxiliary gas heating temperature, 425°C; capillary temperature, 325°C; sheath gas flow rate, 50 psi; auxiliary gas flow rate, 13 psi; and ion spray voltage floating (ISVF) set at -3500 V for negative mode and 3500 V for positive mode. Normalized collision energy for MS/MS was applied in a rolling manner at 20, 40, and 60 eV. The resolution for full MS scans was set to 60,000, while MS/MS scans were set to 7,500. Data acquisition was performed using the Data-Dependent Acquisition (DDA) mode, with a detection mass range of 70–1050 m/z.

### 2.2 Metabolome data analysis

The raw UHPLC-MS data were processed using Progenesis QI software (Waters, Milford, USA) through a series of steps, including baseline filtering, peak identification, peak integration, retention time correction, and peak alignment. A data matrix containing sample names, m/z values, retention times, and peak intensities was generated for further analysis. Metabolite identification was performed by querying databases such as HMDB (http://www.hmdb.ca/), Metlin (https://metlin.scripps.edu/), and the self-compiled Majorbio Database (MJDB) from Majorbio Biotechnology Co., Ltd. (Shanghai, China). The annotated data matrix was uploaded to the Majorbio cloud platform (https://cloud.majorbio.com) for analysis.

Data preprocessing included retaining metabolic features detected in at least 80% of the samples in each group. Missing values in the data matrix were imputed with the minimum value, and each metabolic feature was normalized to its sum. To minimize errors from sample preparation and instrument variability, response intensities of the mass spectrometry peaks were normalized using the sum normalization method. Quality control (QC) samples with a relative standard deviation (RSD) >30% were excluded, and the data were log10-transformed to create the final normalized data matrix for subsequent analyses.

Principal Component Analysis (PCA) and Orthogonal Partial Least Squares Discriminant Analysis (OPLS-DA) were conducted using the R package “ropls” (Version 1.6.2) to evaluate data distribution and model stability, employing a 7-cycle interactive validation process. Differential metabolites were identified based on the OPLS-DA Variable Importance in Projection (VIP) scores (VIP >1) and statistical significance (*p* < 0.05) calculated using Student’s *t*-test (Abouseif et al., 2025).

Significantly altered metabolites between groups were mapped to biochemical pathways using the KEGG database (http://www.genome.jp/kegg/). Metabolites were classified based on their associated pathways or biological functions. Metabolic enrichment analysis was performed to assess whether specific groups of metabolites were overrepresented within functional nodes, transitioning from single-metabolite to group-based annotation. The enrichment analysis was conducted using Python’s “scipy.stats” package (https://docs.scipy.org/doc/scipy/), identifying the most relevant biological pathways associated with experimental treatments.

### 2.3 Transcriptomic data analysis

#### 2.3.1 RNA extraction

Total RNA was extracted from the tissue using TRIzol^®^ Reagent according to the manufacturer’s instructions. Then, RNA quality was determined using a 5300 Bioanalyzer (Agilent) and quantified with an ND-2000 (NanoDrop Technologies). Only high-quality RNA sample (OD260/280 = 1.8~2.2, OD260/230≥2.0, RIN≥6.5, 28S:18S≥1.0, >1μg) was used to construct sequencing library.

#### 2.3.2 Library preparation and sequencing

The RNA purification, reverse transcription, library construction, and sequencing were performed at Shanghai Majorbio Bio-pharm Biotechnology Co., Ltd. (Shanghai, China) according to the manufacturer’s instructions (Illumina, San Diego, CA). The melon RNA-seq transcriptome library was prepared following Illumina^®^ Stranded mRNA Prep, Ligation from Illumina (San Diego, CA) using 1μg of total RNA. Shortly, messenger RNA was isolated according to the polyA selection method by oligo(dT) beads and then fragmented with the fragmentation buffer first. Secondly, double-stranded cDNA was synthesized using a SuperScript double-stranded cDNA synthesis kit (Invitrogen, CA) with random hexamer primers (Illumina). Then the synthesized cDNA was subjected to end-repair, phosphorylation, and ‘A’ base addition according to Illumina’s library construction protocol. Libraries were size selected for cDNA target fragments of 300 bp on 2% Low Range Ultra Agarose followed by PCR amplified using Phusion DNA polymerase (NEB) for 15 PCR cycles. After quantification by Qubit 4.0, the paired-end RNA-seq sequencing library was sequenced with the NovaSeq 6000 sequencer (2 × 150bp read length).

#### 2.3.3 Quality control and read mapping

The raw paired end reads were trimmed and quality controlled by fastp (Langmead and Salzberg, 2012) with default parameters. Then, clean reads were separately aligned to the *C. melo* (melo-DHL92) reference genome with orientation mode using HISAT2 (Langmead and Salzberg, 2012) Software StringTie assembled the mapped reads of each sample in a reference-based approach. The RNA sequencing data were submitted to the National Center for Biotechnology Information (NCBI) under BioProject: PRJNA1394750.

#### 2.3.4 Differential expression analysis and functional enrichment

To identify DEGs (differential expression genes) between two different samples, the expression level of each transcript was calculated according to the transcripts per million reads (TPM) method. RSEM was used to quantify gene abundances. Essentially, differential expression analysis was performed using the DESeq2 (Ateeq et al., 2023). DEGs with |log2FC|≧1 and FDR≤ 0.05(DESeq2) were considered significantly different expressed genes in grafting combinations relative to the self-grafted control fruits. In addition, functional-enrichment analysis including GO and KEGG were performed to identify which DEGs were significantly enriched in GO terms and metabolic pathways at Bonferroni-corrected *P*-value ≤0.05 compared with the whole-transcriptome background. GO functional enrichment and KEGG pathway analysis were carried out by Goatools and KOBAS (Ateeq et al., 2025), respectively.

To validate the RNA sequencing data, we selected eight genes for quantitative reverse transcription PCR (qRT-PCR) analysis. Total RNA was isolated from the fruit pulp of both the grafting combinations and the self-grafted controls at the maturation stage. Using cDNA as a template, qRT-PCR was performed on a Bio-Rad ABI7500 system with SYBR Green detection (TaKaRa). The Tublin gene served as an internal control. The relative expression levels of the target genes were calculated using the 2–ΔΔCT method (Livak and Schmittgen, 2001), with primer sequences provided in **Supplementary Table 1**.

### 2.4 Statistical analysis

All physiological, biochemical, and qRT-PCR data were analyzed using Statistix 8.1. Transcriptome and metabolomic data were processed on the Majorbio I-Sanger Cloud Platform. Results for each parameter are presented as the mean of three biological replicates. The effect of rootstocks on melon fruit biochemical parameters was assessed by one-way analysis of variance (ANOVA), and treatment means were compared using the least significant difference (LSD) test at a significance level of *P* < 0.05.

## 3 Results

### 3.1 Physico-chemical characterization of grafted melons fruit

**Figure 1:**
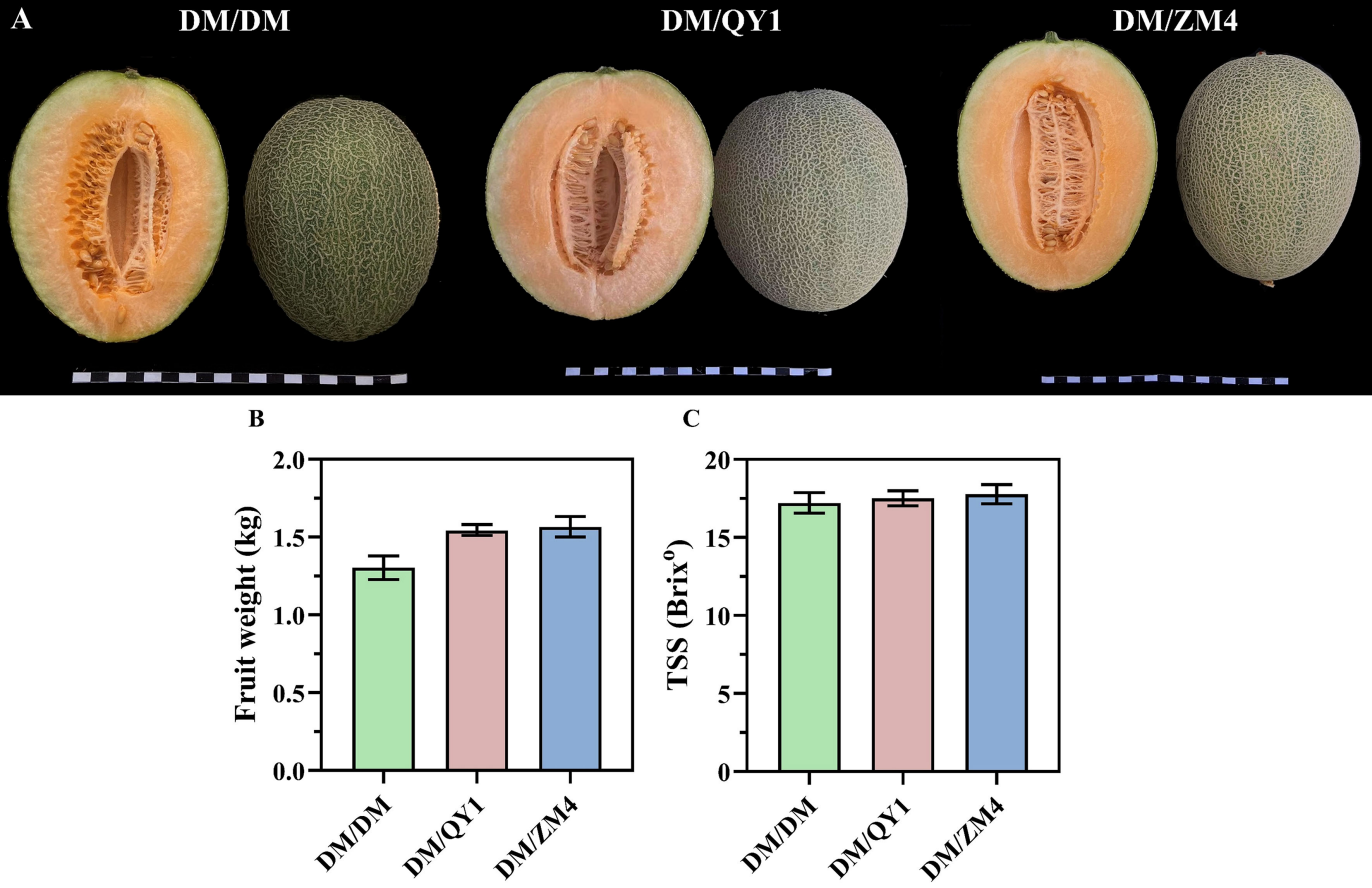
**(A)** Phenotype of melons grafted melon fruits. Scale bar=20cm. **(B)** The quantification of fruit weight in grafted melon fruits. **(C)** The quantification of total soluble solids (TSS) in grafted melon fruits. Different lowercase letters on top of bars indicate significant differences at *P ≤* 0.05.

To evaluate rootstock effects on fruit quality, melon plants were grafted onto two distinct rootstocks, with the resulting fruit phenotypes illustrated in (**Figure 1A**). Fruit weight and total soluble solids (TSS) were measured for fruits harvested from plants grafted onto QY1 and ZM4 rootstocks and self-grafted (control) plants. Melon fruits grafted onto the ZM4 rootstock demonstrated a increase in weight, reaching 1.59 kg, which was substantially greater than that of fruits from either the QY1 rootstock or self-grafted plants (**Figure 1B**). Conversely, melons grafted onto the ZM4 rootstock also exhibited a higher TSS value (17.70 °Brix) compared to all other grafting combinations (**Figure 1C**).

### 3.2 Rootstock-induced transcriptional changes and enriched KEGG pathways in melon fruit

**Figure 2:**
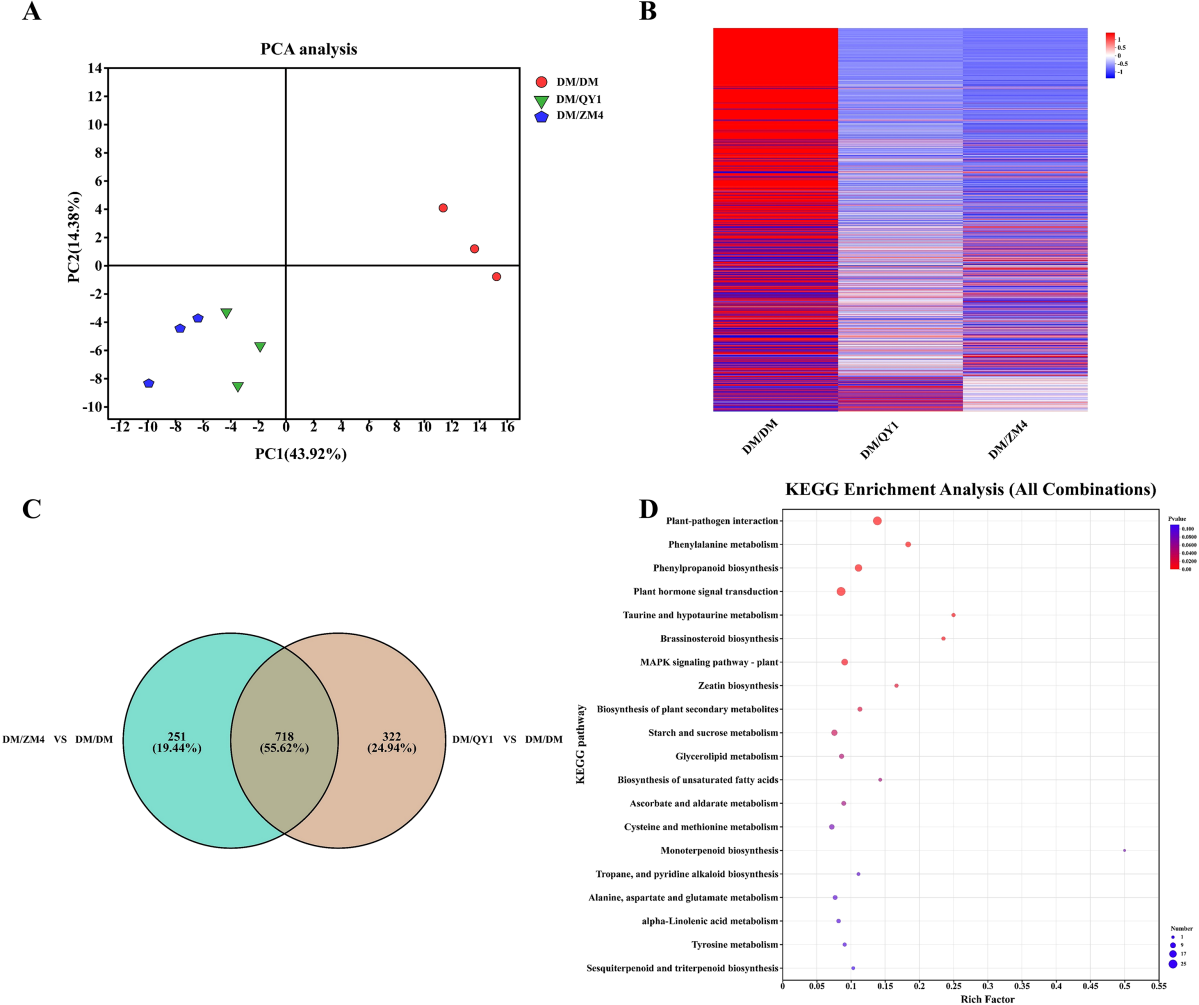
**(A)** Principal component analysis (PCA) of transcriptomic data. **(B)** Heat map showing the DEGs (DM/QY1 vs DM/DM, DM/ZM4 vs DM/DM) expression levels with absolute log2 fold change in grafted melon fruits. The color indicates the relative levels of genes from low (blue) to high (red). **(C)** Venn diagram illustrating the overlap and unique differentially expressed genes (DEGs) between the different comparisons. **(D)** Kyoto Encyclopedia of Genes and Genomes (KEGG) enrichment analysis of DEG’s in the melon fruits in all sample comparison. KEGG enrichment analysis of the DEGs involved in top 20 pathways. The horizontal axis in the plot displays the rich factor to the number of annotated genes, while the vertical axis represents the name of the pathway. The size of the bubble indicates the number of DEGs were found in the relevant pathway. The padjust-values are indicated by colors in bubbles. When the padjust-value is less than 0.05, the route is considered enriched.

A comprehensive transcriptomic analysis was performed on nine fruit tissue libraries to investigate the influence of two rootstocks on quality-related traits in grafted melon scion fruit. Following read refinement and adapter trimming, 57.25 Gb of clean data was generated with a Q30 score >95.52% (**Supplementary Table 2**). Through principal component analysis (PCA) (**Figure 2A**), the samples exhibited a clear trend of intergroup separation in the PCA space, indicating that the rootstock grafting treatment had a significant impact on melon gene expression. Heatmap analysis results (**Figure 2B**) visually displayed the differential patterns of gene expression among different grafting combinations, with the DM/ZM4 group showing broader regions of highly expressed genes, further supporting the specific regulation of the scion transcriptome by the rootstock. Differential expression analysis (**Figure 2C**) revealed that 251 specifically expressed genes were identified in the DM/ZM4 vs. DM/DM comparison group, accounting for 19.44% of the total differential genes; 322 specific genes (24.94%) were found in the DM/QY1 vs. DM/DM comparison group; while 718 differentially expressed genes (55.62%) were shared between the two groups. This result indicates that different rootstocks induced distinct transcriptional reprogramming, with the ZM4 rootstock potentially affecting the physiological processes of the scion by specifically regulating the expression of certain genes.

KEGG enrichment analysis (**Figure 2D**) revealed that multiple pathways related to secondary metabolism played key roles in the growth, development, and stress defense of melon. Core pathways such as phenylpropanoid biosynthesis, monoterpenoid biosynthesis, sesquiterpenoid and triterpenoid biosynthesis are involved in the synthesis of bioactive compounds, which have important functions in plant defense, aromatic properties, and medicinal value (Li et al., 2023). Furthermore, pathways related to amino acid metabolism (e.g., cysteine and methionine metabolism, alanine, aspartate, and glutamate metabolism) are crucial for protein synthesis. Plant hormone-related pathways (particularly plant hormone signal transduction and brassinosteroid biosynthesis) regulate key biological processes such as cell division, stress responses, and growth and development. Starch and sucrose metabolism, along with glycerolipid metabolism, collectively participate in energy storage and cell membrane construction, ensuring normal cellular function under varying environmental conditions.

**Figure 3:**
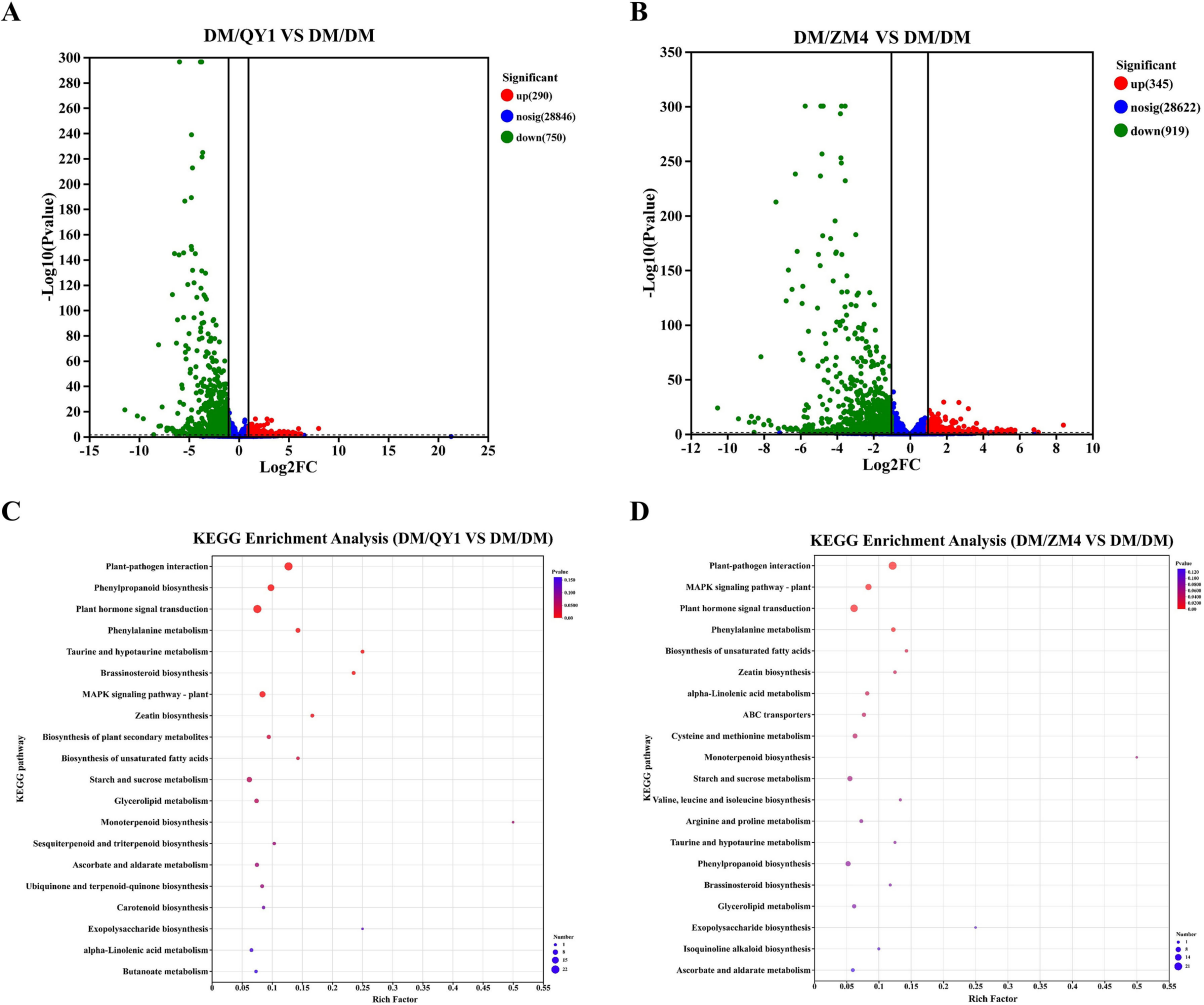
**(A)** Volcano plot showing the differentially expressed genes (DEGs) between DM/QY1 and DM/DM **(B)** Volcano plot depicting the DEGs between DM/ZM4 and DM/DM, with significant genes indicated in red (upregulated), blue (no significant change), and green (downregulated) **(C)** KEGG enrichment analysis for DEGs from the DM/QY1 vs. DM/DM comparison. **(D)** KEGG enrichment analysis for DEGs from the DM/ZM4 vs. DM/DM comparison. The horizontal axis in the plot displays the rich factor to the number of annotated genes, while the vertical axis represents the name of the pathway. The size of the bubble indicates the number of DEGs were found in the relevant pathway. The padjust-values are indicated by colors in bubbles. When the padjust-value is less than 0.05, the route is considered enriched.

The volcano plots of differentially expressed genes (DEGs) clearly illustrate the differences in gene expression under the two grafting comparison conditions (**Figures 3A, B**). In the “DM/QY1 vs DM/DM” comparison group (**Figure 3A**), a total of 1,040 significantly differentially expressed genes were detected, comprising 290 upregulated genes, 750 downregulated genes, and 28,846 non-significant (nosig) genes. The number of downregulated genes was significantly higher than that of upregulated genes, suggesting that the QY1 rootstock may exert a broad suppressive effect on the scion’s expression profile. In the “DM/ZM4 vs DM/DM” comparison group (**Figure 3B**), 1,264 significantly differentially expressed genes were identified, including 345 upregulated genes, 919 downregulated genes, and 28,622 non-significant genes. This reflects the varying degrees of influence different rootstocks have on the gene expression of the scion, providing important clues for further elucidating the molecular mechanisms by which rootstocks regulate the physiology of the scion.

KEGG pathway enrichment analysis results (**Figures 3C, D**) systematically compare the biological pathways affected at the transcriptomic level in melons grafted with different rootstocks (DM/QY1 and DM/ZM4) versus the self-grafted control (DM/DM). Notable divergences included DM/QY1 uniquely enriching sesquiterpenoid and triterpenoid biosynthesis, carotenoid biosynthesis, and ubiquinone/terpenoid-quinone biosynthesis (**Figure 3C**), suggesting enhanced antioxidant and photoprotective responses. Conversely, DM/ZM4 specifically enriched ABC transporters, cysteine and methionine metabolism, branched-chain amino acid biosynthesis, and arginine and proline metabolism (**Figure 3D**), indicating a prioritization of nutrient transport, sulfur amino acid metabolism, and alkaloid-mediated defense. In summary, both rootstocks were capable of activating defense responses and secondary metabolic pathways in the scion, but showed clear differences in specific pathway preferences and regulatory intensities, providing important clues for in-depth analysis of the metabolic basis underlying rootstock-scion interactions.

### 3.3 GO enrichment analysis of all grafting combinations

**Figure 4:**
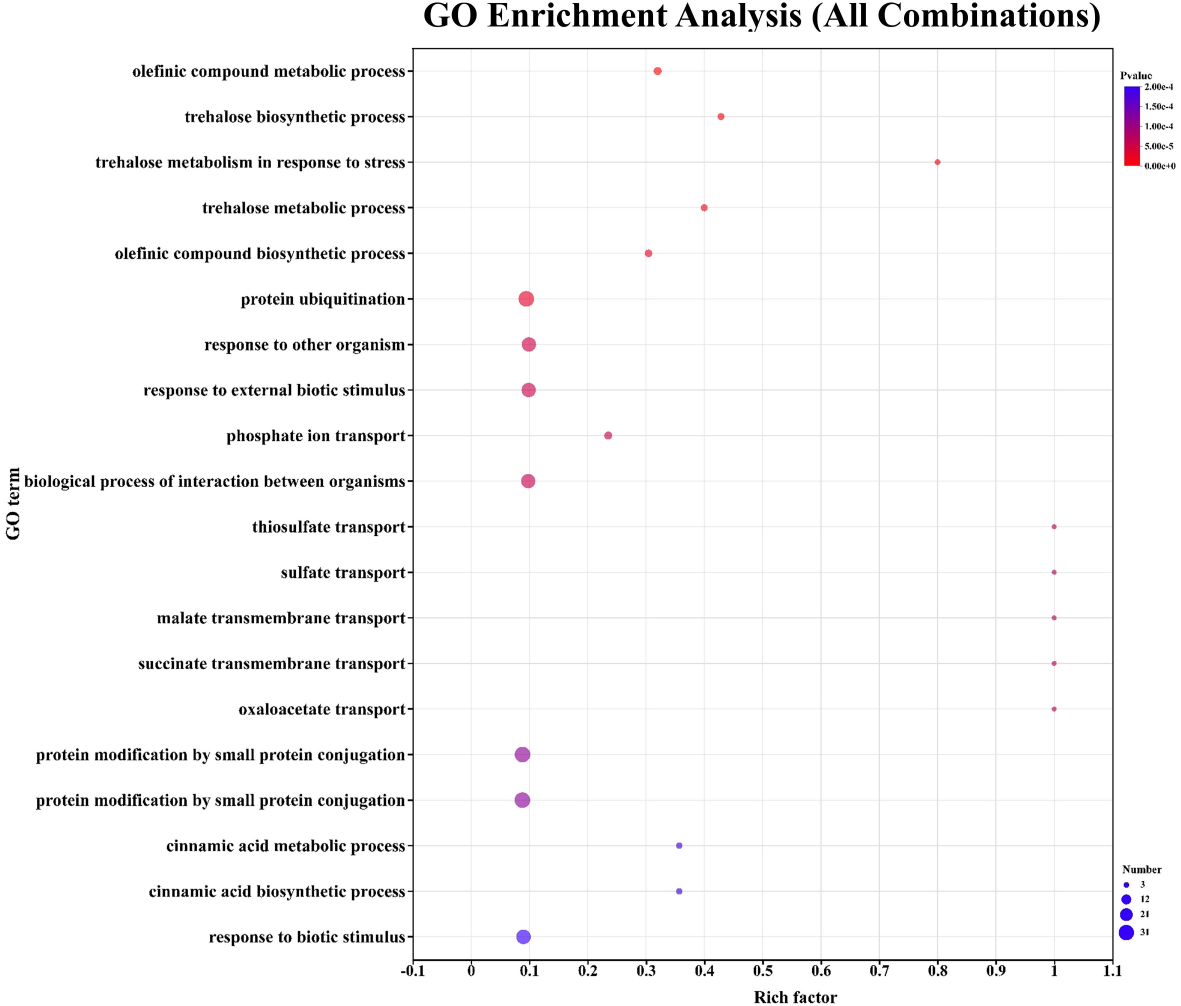
Gene Ontology (GO) enrichment analysis of differentially expressed genes in all grafting combinations. The horizontal axis in the plot displays the rich factor to the number of annotated genes, while the vertical axis represents the biological process name. The size of the bubble indicates the number of DEGs were found in the relevant biological process. The *p*-values are indicated by colors in bubbles. When the *p*-value is less than 0.05, the biological function is considered enriched.

Gene Ontology (GO) enrichment analysis of RNA-seq data revealed that phosphate ion transport was significantly enriched biological process, indicating its central role in cellular energy metabolism and signal transduction (**Figure 4**). Through this GO enrichment analysis (all combinations), we identified significant enrichment in multiple biological processes in the samples, covering metabolism, transport, protein modification, interspecies interactions, and stress responses. The trehalose biosynthetic process and tre-halose metabolism were also enriched, suggesting that plays key roles in the regulation of carbon allocation and stress adaptation (Mollavali and Bornke, 2022).The enrichment of cinnamic acid metabolic process and cinnamic acid biosynthetic process suggests the potential presence of biosynthesis or metabolic activities related to cinnamic acid in the samples, which may be associated with secondary metabolism or defense mechanisms in plants (Gómez-Martínez et al., 2021).

### 3.4 Metabolism analysis

**Figure 5:**
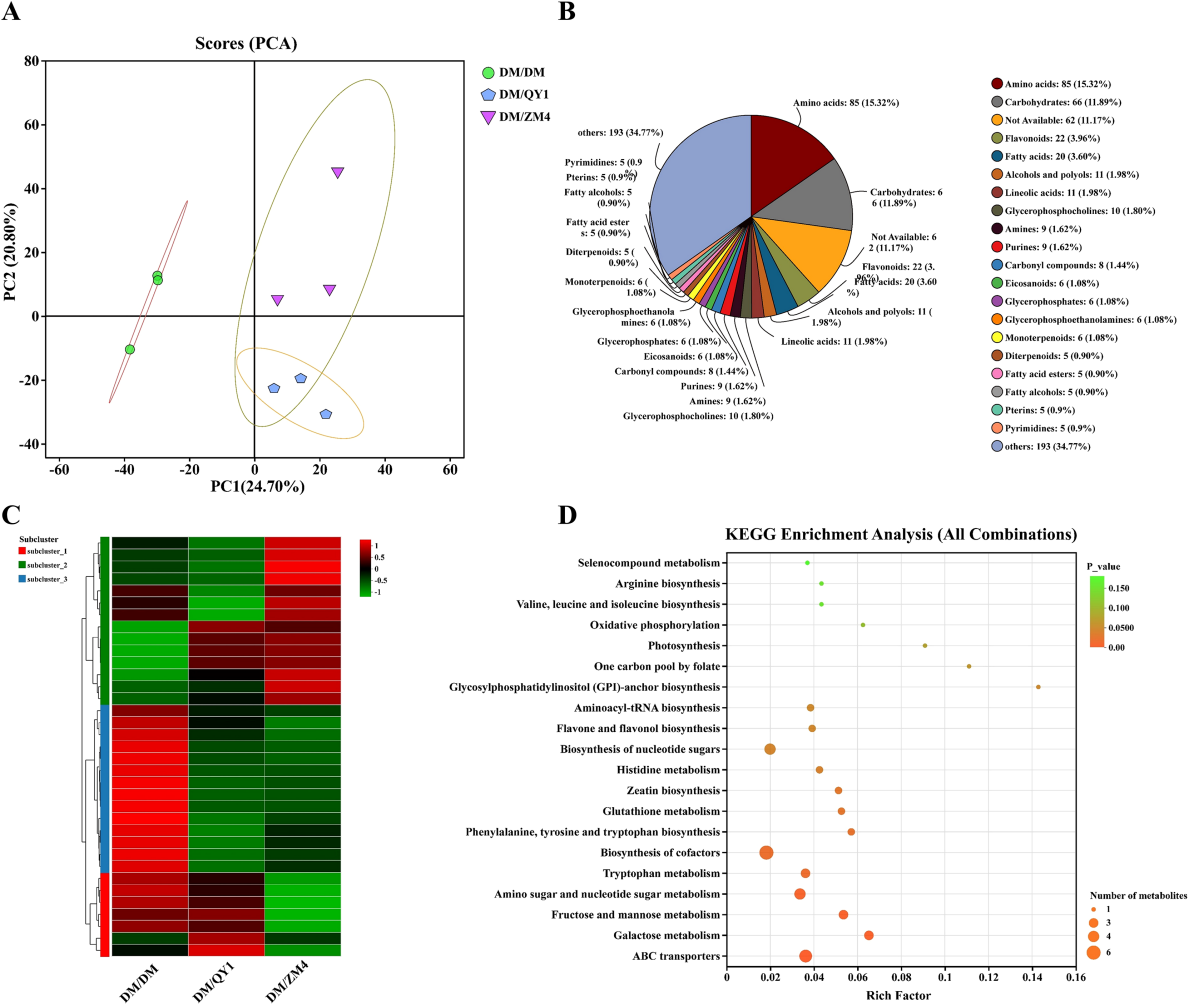
**(A)** Principal component analysis (PCA) of metabolite data. **(B)** Pie chart illustrating the proportion of different metabolite classes in grafted melon samples with VIP > 1 in melon fruit. **(C)** Heatmap of differentially expressed metabolites across all grafting combinations. **(D)** KEGG enrichment analysis for differentially expressed metabolites across grafted melon combinations. Each circle in the plot represents the number of associated metabolites and is position according to its rich factor. The *p-*values are indicated by colors; color change from red to blue indicate 0 to 0.05 *p*-value.

Principal component analysis (PCA) is performed on different samples, which shows the distribution pattern of samples in a score plot. The first principal component (PC1) of PCA explains 24.70% of the variance, while the second principal component (PC2) explains 20.80% (**Figure 5A**). As shown in the figure, the samples from different treatment groups exhibit a distinct clustering trend in the PCA space, indicating that the treatments have a significant impact on the metabolic spectrum of the samples. For example, a clear distinction exists between the DM/QY1 and DM/ZM4 groups, suggesting that these treatments may have led to significant changes in the metabolic profiles of the samples. A pie chart details the relative abundance of different metabolite classes in the sample (**Figure 5B**). Among them, amino acids are the most relatively abundant, accounting for 85 species (15.32%), followed by carbohydrates, 66 species (11.89%), fats, 3.60%, and flavonoids, 22 species (3.96%).

Integrated metabolomic analysis revealed core KEGG pathways central to the observed metabolic phenotype (**Figure 5D**): phenylalanine, tyrosine and tryptophan biosynthesis, essential for aromatic amino acid supply and precursor roles; biosynthesis of cofactors, underpinning enzymatic activities across metabolism; amino sugar and nucleotide sugar metabolism, crucial for glycan and cell wall component synthesis; fructose and mannose metabolism, a key entry point for glycolytic energy production and hexose interconversion; and glutathione metabolism, indicative of active redox homeostasis and detoxification processes. Furthermore, significant enrichment of ABC transporters highlights their critical role in the cellular uptake of nutrients (potentially including sugars, amino acids, and cofactors) and efflux of metabolites or xenobiotics, thereby directly supporting and regulating the flux through these core metabolic networks. Other compounds with relatively high enrichment include tryptophan, tryptophan, zeatin biosynthesis, histidine, and flavone and flavonol biosynthesis.

**Figure 6:**
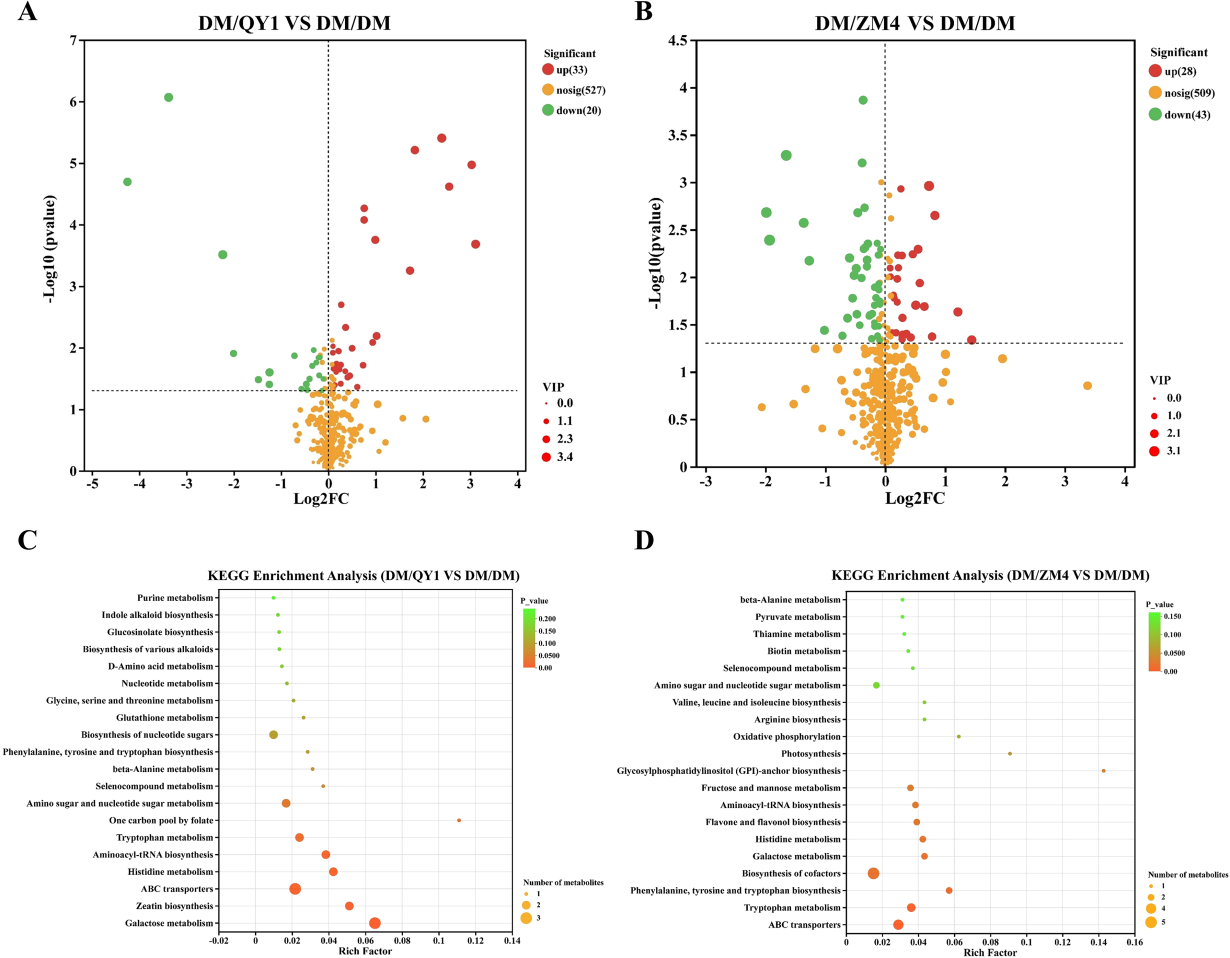
**(A)** Volcano plot showing significant upregulated (green) and downregulated (red) differentially accumulated metabolites in DM/QY1 vs. DM/DM. **(B)** Volcano plot illustrating significant upregulated (green) and downregulated (red) metabolites in DM/ZM4 vs. DM/DM. **(C)** KEGG enrichment analysis for DEGs in DM/QY1 vs. DM/DM. **(D)** KEGG enrichment analysis for DEGs in DM/ZM4 vs. DM/DM. Each circle in the plot represents the number of associated metabolites and is position according to its rich factor. The p-values are indicated by colors.

By comparing the differences in metabolite expression profiles between the two rootstock grafting treatments (DM/QY1 and DM/ZM4) and the self-grafted control (DM/DM) (**Figures 6A, B**), it was found that the DM/ZM4 treatment (**Figure 6B**) induced more pronounced metabolic reprogramming, with a total of 71 differentially expressed metabolites identified, predominantly down-regulated (43 down, 28 up). In contrast, the DM/QY1 treatment (**Figure 6A**) resulted in 53 differentially expressed metabolites, showing a main trend of up-regulation (33 up, 20 down). These results indicate that different rootstocks can specifically reconfigure the metabolic network of the scion. KEGG enrichment analysis revealed that metabolic pathways such as phenylpropanoid biosynthesis, flavonoid biosynthesis, and ABC transporters were significantly enriched in the DM/QY1 group (**Figure 6C**), indicating that grafting the melon cultivar DuMi 5 (DM) onto the pumpkin rootstock QY1 may significantly influence the synthesis and transport of secondary metabolites, which is likely associated with enhanced resistance of the grafted plants. In the DM/ZM4 group (**Figure 6D**), pathways including phenylpropanoid biosynthesis, phenylalanine metabolism, and glutathione metabolism also showed significant enrichment, suggesting that this rootstock may function by regulating the biosynthesis of antioxidant substances in melon. Further analysis demonstrated that in the DM/QY1 vs. DM/DM comparison, significantly enriched pathways mainly included purine metabolism, indole alkaloid biosynthesis, glucosinolate biosynthesis, and the biosynthesis of phenylalanine, tyrosine, and tryptophan, indicating the activation of amino acid metabolism and secondary metabolite synthesis pathways. In contrast, the DM/ZM4 vs. DM/DM comparison exhibited significant enrichment in β-alanine metabolism, pyruvate metabolism, and branched-chain amino acid (valine, leucine, isoleucine) biosynthesis, highlighting a notable regulatory effect on energy metabolism. These results demonstrate that the two rootstocks induce distinct metabolic reprogramming processes: DM/QY1 primarily influences secondary metabolites and amino acid metabolism, while DM/ZM4 focuses more on energy metabolism and branched-chain amino acid metabolism.

### 3.5 Rootstock-driven reprogramming of amino acid metabolism in melon scions

**Figure 7:**
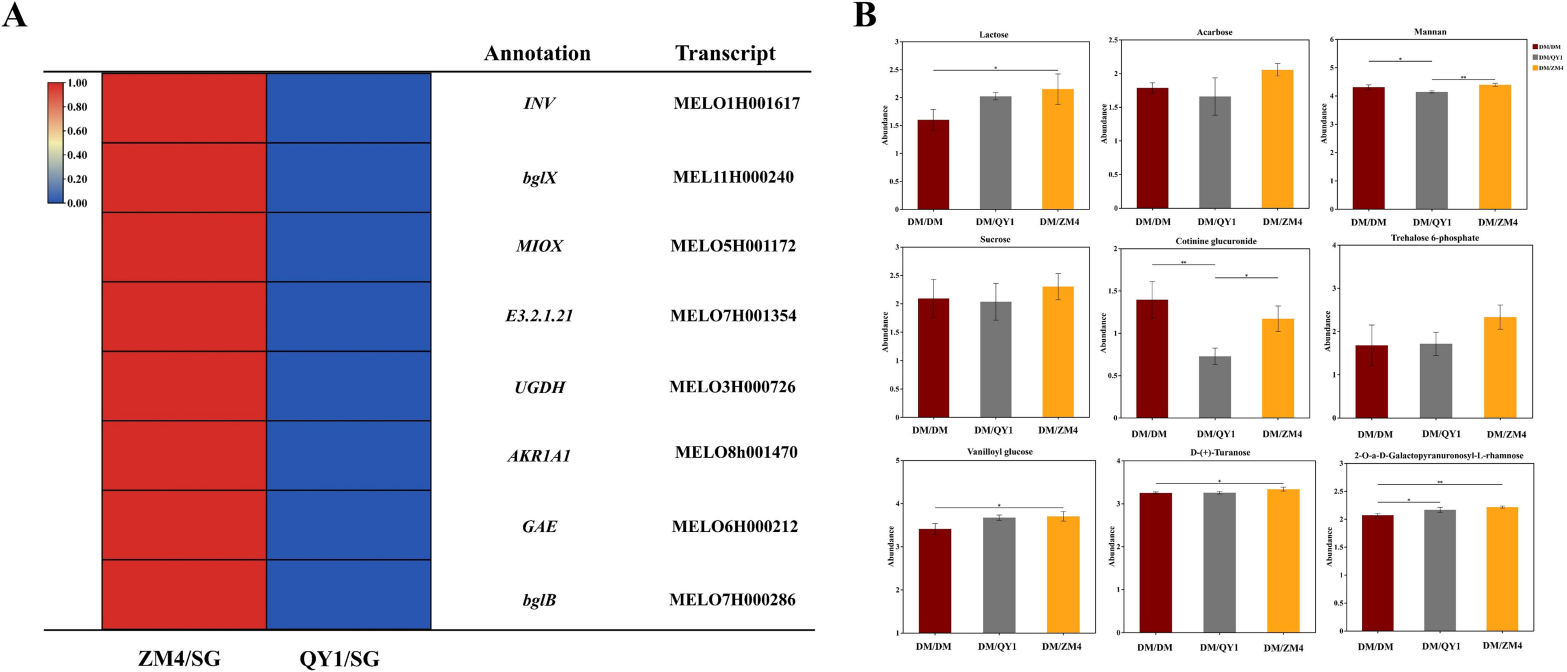
**(A)** Heatmap showing amino acid gene expression levels of selected transcripts in melon grafted onto ZM4 and QY1 rootstocks. **(B)** Bar charts representing the amino acid levels of key metabolites in melon fruits grafted onto ZM4 and QY1 rootstocks. Relative abundance of amino acids contents was compared to the internal standard according to *post-hoc* test at *P* ≤ 0.05.

Transcriptomic data (**Figure 7A**) shows that ZM4/SG significantly down regulates gene, whereas QY1/SG induces. Metabolomic validation (**Figure 7B**) reveals corresponding changes:ZM4/SG accumulates phenylpropanoid intermediates L-Tyrosin, Tryptophol and, sulfur metabolites (N-StearoylArginine), aligning with PAL-mediated flux diversion and cysE-dependent acetyl-CoA competition.QY1/SG enhances TCA cycle derivatives (L-Leucine; Glycyl-prolyl-arginyl-valyl-valyl-glutamic acid) consistent with MDH2-supported carbon skeleton supply and *EC1.14.17.4* activity. Crucially, ZM4 rootstock promotes phenylalanine-to-tyrosine conversion (negative PAL-phenylalanine correlation), while QY1 accelerates glutamate decarboxylation (*GAD2*) and GABA synthesis. These rootstock-specific strategies optimize nitrogen allocation: ZM4 favors secondary metabolite production, whereas QY1 enhances energy metabolism and peptide biosynthesis.

### 3.6 Rootstock-driven reprogramming of sugar metabolism in melon scions

**Figure 8:**
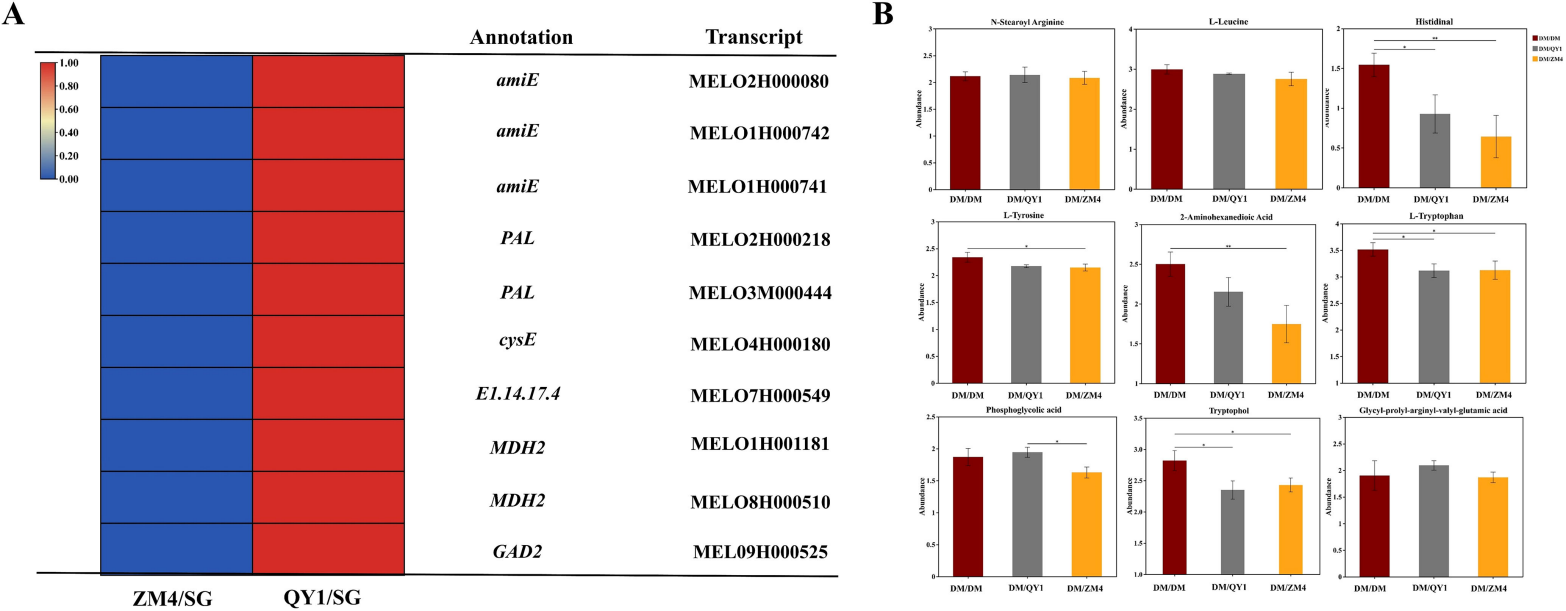
**(A)** Heatmap showing levels of selected sugar related transcripts in melon grafted onto ZM4 and QY1 rootstocks. **(B)** Bar charts representing the sugar levels of key metabolites in melon fruits grafted onto ZM4 and QY1 rootstocks. Relative abundance of sugar contents was compared to the internal standard according to *post-hoc* test at *P* ≤ 0.05.

Transcriptomic profiling (**Figure 8A**) demonstrated these genes are highly expressed in melon scions grafted onto *Cucumis metuliferus* (ZM4/SG) rootstocks. ZM4/SG exhibited significant upregulation of Sucrose-cleaving enzymes: *INV* and *bglX/bglB* (β-glucosidases, 2.3–3.0-fold, P<0.01) (**Figure 8B**). Nucleotide-sugar synthases: *UGDH* (UDP-glucose dehydrogenase) and *GAE* (UDP-glucuronate epimerase), driving expansion of UDP-sugar precursors for glycosylation. Concomitant metabolomic shifts (**Figure 8B**) confirmed ZM 4/SG-specific metabolic advantages: Enhanced anabolic flux: Accumulation of cell wall polymers (mannan) and glycosides (vanilloyl glucose; cotinine glucuronide), directly linked to *UGDH/GAE* activity. Stress resilience: *AKR1A1* induced elevated xylitol, While *MIOX* supported redox homeostasis, consistent with trehalose-6-phosphate accumulation. Conversely, QY1/SG showed compromised metabolism: Suppressed E3.2.1.31 (β-glucuronidase) reduced free glucuronic acid, limiting glycoside hydrolysis. Diminished MIOX expression constrained NADPH regeneration from myo-inositol. Biological implication: ZM4 rootstock orchestrates coordinated induction of hydrolytic (*INV/bglX*), biosynthetic (*UGDH/GAE*), and stress-adaptive (*AKR1A1/MIOX*) genes, optimizing carbon flux toward both structural polysaccharides and high-value glycosides (**Figures 8A, B**).

**Figure 9:**
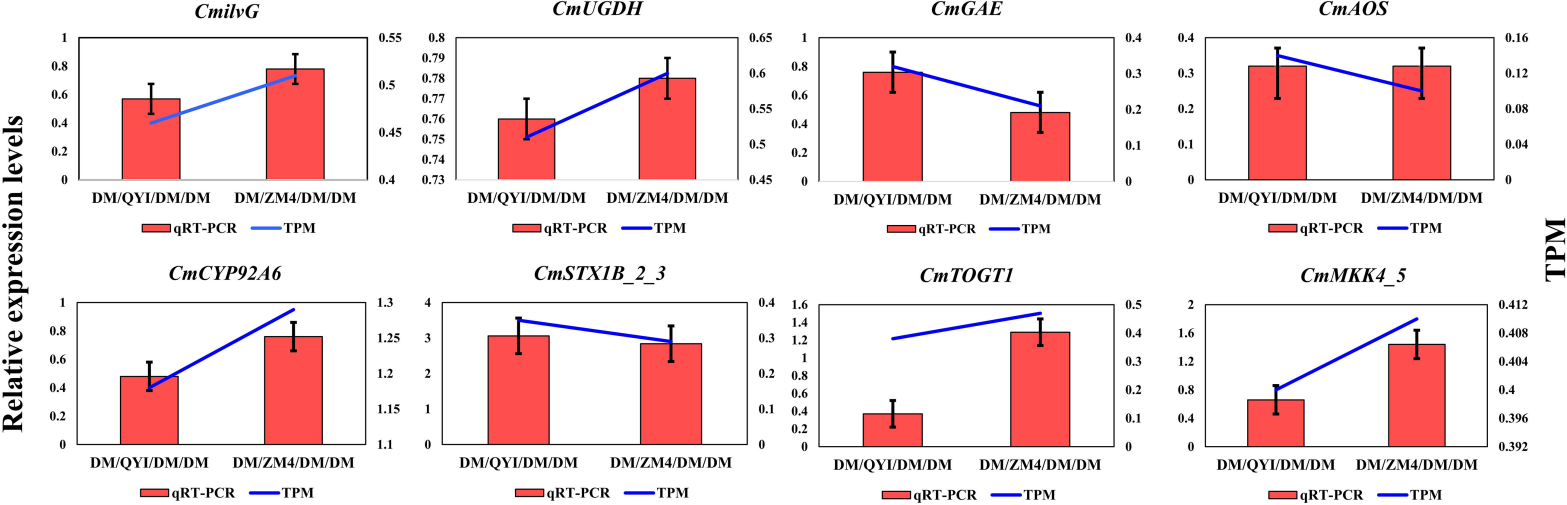
Confirmation of RNA-sequencing data using quantitative reverse transcription polymerase chain reaction (qRT‐PCR) analysis. The expression profile of randomly selected eight genes verified by qRT‐PCR in two rootstocks grafted melon fruit. The y‐axis shows the qRT‐PCR (red histogram) and RNA- seq TPM values (blue line) while x‐axis indicates samples from QY1 and ZM4 rootstock grafted melon fruits as compared to self-grafted (DM/DM).

To validate our transcriptomic findings, the expression of eight randomly selected genes was analyzed by qRT-PCR. The results showed a consistent expression pattern with the RNA-seq data, confirming the high reliability of our sequencing analysis (**Figure 9**).

## 4 Discussion

The current study diligently unravels the intricate molecular mechanisms underpinning quality attributes related to sugars and amino acids in melons *(Cucumis melo L.*) grafted onto two distinct rootstocks. Through integration of comprehensive transcriptomic and metabolomic analyses, we have pinpointed rootstock-specific strategies. These strategies prove remarkably effective in optimizing carbon allocation, channeling it towards the synthesis of structural polysaccharides and high-value glycosides. This precise redirection is a direct outcome, significantly elevating fruit quality and bolstering the melons’ stress resilience.

### 4.1 ZM4 rootstock enhances melon flavor through activation of carbohydrate metabolism pathways

The beneficial and detrimental impacts of grafting on sugar - related sensorial quality attributes of fruits have been well - documented in various Cucurbitaceae crops (Kyriacou et al., 2017). Previous research has indicated that certain rootstock combinations can significantly elevate sugar content compared to non - grafted plants (Kaleem et al., 2025). In this study, we found that the sugar content in fruits grafted onto the ZM4 rootstock was higher than in those grafted onto the QY1 rootstock, and both were higher than in self-grafted fruits (**Figure 1C**). Our results demonstrate that pumpkin rootstock (QY1) grafted melon exhibits a decrease in fruit sugar content, a finding consistent with the dual role of source and sink tissues in determining fruit quality traits. While pumpkin rootstock is known to enhance the photosynthetic capacity and light-use efficiency of leaves; thereby increasing photoassimilate production in grafted plants—sucrose accumulation within the fruit ultimately depends also on sink strength and the metabolic conversion of imported photoassimilates (Yang et al., 2020). Prior research indicates that pumpkin rootstock can lead to reduced activity of key sucrose-metabolizing enzymes, such as insoluble acid invertase, sucrose phosphate synthase and sucrose synthase, limiting the conversion of photoassimilates into sucrose in grafted watermelon fruits (Yang et al., 2020). Decrease in soluble sugars can be attributed to vigorous roots of pumpkin rootstock, which act as additional sinks and reduce assimilation flow to fruit and thereby reduce fruit sugar content (Lecholocholo et al., 2022). Devi et al. (2020) found that fruit quality of grafted watermelon was optimized with a 7-day delayed harvest from when those indicators of fruit physiological maturity occurred. The increase in time to harvest is likely due to the increased vigor of the rootstock, including increased water, oxygen, and mineral uptake.

The remarkable increase in glucose and sucrose content in ZM4-grafted melons at ripening represents a fundamental shift in carbohydrate metabolism. The accumulation of glucose and sucrose in grafted watermelon has been associated with genes such as fructose - bisphosphate aldolase 2, fructokinase, sucrose synthase, sucrose - phosphate synthase (SPS), insoluble acid invertase, and the sugar transporter gene SWT3b (Aslam et al., 2020). In citrus grafted onto different rootstocks, differences in soluble sugar are primarily regulated by the expression of sucrose metabolism - related genes, such as sucrose phosphate synthase1, and the activities of SPS and neutral invertase enzymes (Zaman et al., 2025; Ling et al., 2019). Invertase (INV), β-glucosidase (bglX/bglB), and nucleotide sugar synthases (such as UDP-glucose dehydrogenase (UGDH) and UDP-glucuronate epimerase (GAE) play key roles in plant cell wall polysaccharide synthesis and metabolism. Invertase breaks down sucrose into glucose and fructose, providing energy and regulating cell wall component synthesis (Ouyang et al., 2023). β-glucosidase affects polysaccharide degradation and structure, influencing cell wall functionality (Broach et al., 2012; Ouyang et al., 2023). UGDH and GAE promote the synthesis of uronic acid-containing polysaccharides, ensuring proper cell wall assembly and function (Broach et al., 2012; Ouyang et al., 2023). The ZM4 rootstock significantly upregulated sucrose - cleaving enzymes, namely invertase [INV] and β- glucosidases [bglX/bglB], as well as nucleotide - sugar synthases (UDP - glucose dehydrogenase [UGDH] and UDP - glucuronate epimerase [GAE]), which promoted the accumulation of glucose and fructose (**Figures 8A, B**). These enzymatic changes are in line with previous studies showing that rootstocks modulate carbohydrate metabolism through source - sink dynamics (Kaleem et al., 2025; Miao et al., 2019). The ZM4 - mediated induction of UGDH/GAE enzymes expands the UDP - sugar precursor pools. This not only enhances the sweetness derived from glycosides but also promotes the accumulation of cell wall polysaccharides (e.g., mannan) to strengthen the structural integrity of the fruit. This dual metabolic coordination is similar to the reprogramming events observed in Cucurbitaceae graft systems, where rootstocks regulate sink - specific sugar transport and partitioning. Notably, our findings are consistent with studies demonstrating that domesticated melon (*Cucumis melo*) fruits experience a developmental shift from symplastic to apoplastic phloem unloading. This shift is facilitated by sucrose transporters such as *CmSWEET10* and *CmSUT4*, which allow sucrose to be effluxed into the apoplast. Similarly, the ZM4 - driven expansion of UDP - sugar precursors likely facilitates both glycosylation reactions for sweetness enhancement and polysaccharide polymerization for cell wall fortification, analogous to how the cell wall invertase (CwIN) activity in melons directs hexose flux towards either fruit expansion or sucrose accumulation depending on the developmental stage (Zhou et al., 2023). This conserved mechanism emphasizes that UDP - sugar metabolism serves as a pivotal node in coordinating quality traits in sink tissues.

**Figure 10:**
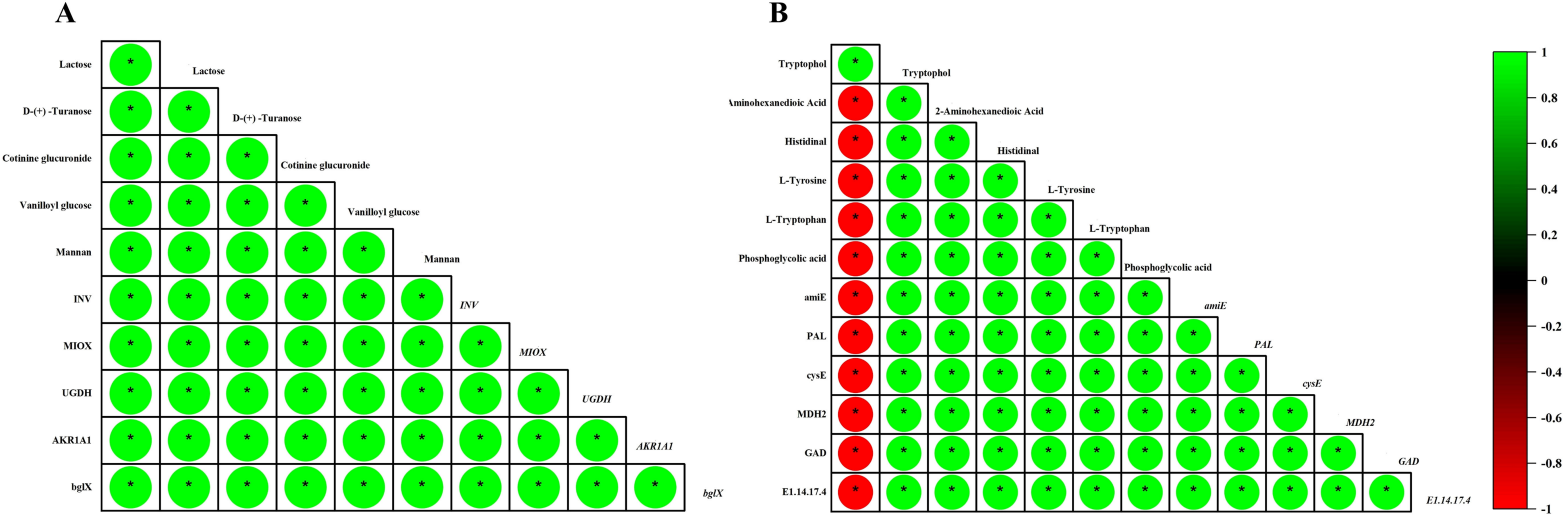
**(A)** Heatmap showing sugar gene expression correlation with metabolites in melon grafted onto ZM4 and QY1 rootstocks. **(B)** Heatmap showing amino acid gene expression correlation with amino acid metabolites in melon grafted onto ZM4 and QY1 rootstocks.

The upregulation of invertase (INV) and β - glucosidases (bglX/bglB) in ZM4 - grafted fruits is a critical regulatory point in sugar metabolism. These enzymes catalyze the hydrolysis of sucrose into glucose and fructose, which can then be utilized in various metabolic processes (Aslam et al., 2020; Huang et al., 2018; Cheng et al., 2017). However, QY1 suppressed glycoside hydrolysis (through reduced β - glucuronidase [E3.2.1.31] expression) and limited NADPH regeneration, suggesting a trade - off between stress adaptation and flavor - related metabolism. Such metabolic prioritization is in agreement with studies on grafted tomatoes, where rootstocks differentially regulate energy allocation under stress (Jenkins et al., 2022) (Liu et al., 2023) (**Figures 10A, B**).

### 4.2 Rootstock-mediated reprogramming of melon fruit quality attributes involves transcriptional and metabolic regulation of amino acid metabolism

The specific rootstock used can considerably affect the levels of different sweetness compounds and the overall fruit quality (Maoz et al., 2022). For instance, Tianzhen No. 1 rootstock demonstrably reduces levels of bitter amino acids (leucine, isoleucine, valine) compared to alternatives like Sizhuang No. 12 (Kaleem et al., 2025). Crucially, aromatic amino acids serve as pivotal aroma precursors: L-Tyrosine enrichment fuels phenolic and phenylpropanoid volatiles defining melon’s signature fragrance (Huang et al., 2017; Gonda et al., 2010; Alam et al., 2022), while Tryptophan metabolism generates complex indole derivatives that can impart off-flavors upon over-accumulation (Alam et al., 2025; Gonda et al., 2010). Sulfur-containing metabolites (e.g., N-Stearoyl Arginine) contribute desirable sulfury notes at optimal levels but cause unpleasant odors when excessive (Alam et al., 2025; Gonda et al., 2010). Concurrently, upregulated lysine decarboxylase (LDC) and glutamate decarboxylase (GAD) activities link amino acid metabolism to stress tolerance via polyamine and γ-aminobutyric acid (GABA) synthesis (Wang et al., 2025; Yan et al., 2023).

Transcriptomic analysis (**Figure 7A**) revealed that ZM4/SG significantly downregulated specific genes, while QY1/SG induced them. Metabolomic validation (**Figure 7B**) mirrored these changes: ZM4/SG accumulated phenylpropanoid pathway intermediates (L-Tyrosine, Tryptophan) and sulfur metabolites (N-Stearoyl Arginine), aligning with PAL-mediated flux diversion and cysE-dependent competition for acetyl-CoA. In contrast, QY1/SG enhanced levels of TCA cycle derivatives (L-Leucine; Glycyl-prolyl-arginyl-valyl-valyl-glutamic acid) and bile acid hydroxylation products (Taurodeoxycholic acid), consistent with MDH2-supported carbon skeleton supply and EC 1.14.17.4 activity. Crucially, ZM4 rootstock promoted the conversion of phenylalanine to tyrosine (evidenced by a negative correlation between PAL activity and phenylalanine levels), while QY1 accelerated glutamate decarboxylation (via GAD2) and GABA synthesis. These rootstock-specific metabolic strategies optimize nitrogen allocation: ZM4 favors secondary metabolite production linked to fruit quality attributes, whereas QY1 enhances energy metabolism (TCA cycle) and peptide biosynthesis.

### 4.3 Coordinated metabolic reprogramming in ZM4-grafted melons

AKRs, as phase I metabolic enzymes, utilize NADPH or NADH as cofactors to catalyze the reduction reactions of various aldehydes and ketones. This activity aids in detoxifying harmful carbonyl compounds and helps regulate cellular metabolic balance (Andress Huacachino et al., 2024; Mindnich and Penning, 2009; Yu et al., 2020). In our study, ZM4-rootstock grafting significantly induced AKR1A1 expression, which corresponded with elevated xylitol accumulation. MIOX upregulation in ZM4-grafted fruits also suggests a potential shift in inositol metabolism, possibly influencing pathways related to redox homeostasis. MIOX catalyzes the oxidation of myo-inositol to D-glucuronic acid, a precursor for ascorbate (vitamin C) biosynthesis (Khan et al., 2025). Notably, we observed an associated accumulation of trehalose 6-phosphate, a metabolite known from other systems to function in stress response (Khan et al., 2023; Sarkar and Sadhukhan, 2022). While the induction of these markers (AKR1A1, MIOX, trehalose-6-phosphate) is often discussed in the context of abiotic stress adaptation, our study did not include direct stress tolerance assays. Therefore, we interpret their upregulation here primarily as indicators of a significant metabolic reprogramming in grafted fruits, which concurrently enhanced sweetness in melon.

In contrast, QY1 concurrently suppressed glycoside hydrolysis (via reduced β-glucuronidase [EC 3.2.1.31] expression) and constrained NADPH regeneration capacity. This metabolic pattern suggests a potential trade-off in QY1-grafted plants, prioritizing stress adaptation at the expense of flavor-related secondary metabolism. While this study elucidates key mechanisms of rootstock-mediated metabolic reprogramming, several limitations necessitate targeted investigation. Notably, the contribution of phyto-hormonal networks (e.g., auxin-ethylene crosstalk) in coordinating rootstock-scion signaling remains unresolved—systematic hormonal profiling combined with inhibitor/agonist treatments would delineate the signaling hierarchies governing metabolic shifts observed in grafted melons (Ashraf et al., 2025). Furthermore, controlled environment conditions inherently limit extrapolation to commercial production; multi-location field trials under divergent abiotic stressors (drought, salinity, temperature extremes) must validate the ecological robustness of rootstock effects (Yaschenko et al., 2024).

## 5 Conclusion

In conclusion, this study emphasizes the critical role of rootstock selection in regulating melon fruit flavor. Grafting onto the ZM4 and QY1 rootstocks identified distinct fruit flavor-related pathways modulated by the rootstock genotype. The rootstock genotype ZM4 increase the sugar accumulation by activating carbohydrate metabolism pathway in melon fruit. Furthermore, this rootstock is resistant to root-knot nematodes (Zhang et al., 2025), providing a suitable alternative for melon growers, as it not only resists root-knot nematodes but also enhances the melon flavor profile. This rootstock can also serve as a valuable genetic resource for breeding programs aimed at enhancing melon flavor. While this study elucidates key mechanisms of rootstock-mediated metabolic reprogramming, several limitations necessitate targeted investigation. Controlled environment conditions inherently limit extrapolation to commercial production; multi-location field trials under divergent abiotic stressors (drought, salinity, temperature extremes) must validate the ecological robustness of rootstock effects. Addressing these knowledge gaps demands integrated approaches: high-resolution spatiotemporal transcriptomics to resolve developmental regulation, hormone transport imaging to visualize long-distance signaling, and machine learning-driven integration of metabolomic-sensory datasets to establish predictive flavor models. Collaborative efforts across physiology, genomics, and food science will accelerate the development of next-generation rootstocks with precision-tailored impacts on fruit quality.

## Data Availability

The data in this study are accessible in **Supplementary Materials** and Bioproject accession number PRJNA1394750.

## References

[B1] Abouseif Y. Kadeer A. Haishun C. Kaleem M. M. Notaguchi M. Qifan X. . (2025). Integrated multi-omics analysis reveals the molecular mechanism of light intensity-enhanced healing in cotyledon-less splice grafted watermelon. Hortic. Res. 13, uhaf293. doi: 10.1093/hr/uhaf293, PMID: 41726326 PMC12923272

[B3] Alam S. M. Liu D. H. Liu Y. Z. Han H. Hussain S. B. Ateeq M. (2022). Molecular elucidation for the variance of organic acid profile between citrus top and bottom canopy fruits. Sci. Hortic. (Amsterdam). 302, 111181. doi: 10.1016/j.scienta.2022.111181, PMID: 38826717

[B2] Alam S. M. Liu D. Luo Y. Khan M. A. Han H. Zaman F. . (2025). Elevated expression of CsCit and CsALMT9-like plays a key role in lowering citrate and increasing malate accumulation in *Citrus tamurana*× *natsudaidai* ‘Haruka’fruit. Hortic. Plant J. doi: 10.1016/j.hpj.2024.12.003, PMID: 38826717

[B4] Andress Huacachino A. Joo J. Narayanan N. Tehim A. Himes B. E. Penning T. M. (2024). Aldo-keto reductase (AKR) superfamily website and database: An update. Chem. Biol. Interact. 398, 111111. doi: 10.1016/j.cbi.2024.111111, PMID: 38878851 PMC11232437

[B5] Ashraf M. A. Ateeq M. Zhu K. Asim M. Mohibullah S. Riaz T. . (2025). Phytohormone networks orchestrating lateral organ adaptations to hypoxia and reoxygenation in fruit crops. Plant Cell Environ. 49, 1–16. 10.1111/pce.70242 41084125

[B6] Aslam A. Zhao S. Azam M. Lu X. Liu W. (2020). Comparative analysis of primary metabolites and transcriptome changes between ungrafted and pumpkin-grafted watermelon during fruit development. PeerJ 8, e8259. doi: 10.7717/peerj.8259, PMID: 31934503 PMC6951286

[B7] Ateeq M. Khan A. H. Zhang D. Alam S. M. Shen W. Wei M. . (2023). Comprehensive physio-biochemical and transcriptomic characterization to decipher the network of key genes under waterlogging stress and its recuperation in *Prunus persica*. Tree Physiol. 43, tpad029. doi: 10.1093/treephys/tpad029, PMID: 36905330

[B8] Ateeq M. Zhang D. Xiao J. Zhang H. Shen X. Meng J. . (2025). Decoding submergence tolerance in Prunus persica: Integrated transcriptomic and metabolomic acclimations of antioxidant system, cell wall dynamics, and hormonal signaling. Hortic. Adv. 3, 5. doi: 10.1007/s44281-024-00058-z, PMID: 30311153

[B9] Bie Z. Peng Y. Kaleem. M. ,. M. Wei L. Geng S. Wang L. (2025). Grafting as a tool for improving growth and stress tolerance in vegetable crops BT - growth regulation and quality improvement of vegetable crops: physiological and molecular features. Eds. Ahammed G. J. Zhou J. ( Springer Nature Singapore), 587–619.

[B10] Bie ZhiLong B. Z. Nawaz M. A. Huang Yuan H. Y. Lee JungMyung L. J. Colla G. (2017). Introduction to vegetable grafting., in: Vegetable Grafting: Principles and Practices ( CABI Wallingford UK), 1–21.

[B11] Broach B. Gu X. Bar-Peled M. (2012). Biosynthesis of UDP-glucuronic acid and UDP-galacturonic acid in *Bacillus cereus* subsp. cytotoxis NVH 391-98. FEBS J. 279, 100–112. doi: 10.1111/j.1742-4658.2011.08402.X, PMID: 22023070 PMC3240692

[B12] Camalle M. D. Pivonia S. Zurgil U. Fait A. Tel-Zur N. (2023). Rootstock identity in melon-pumpkin graft combinations determines fruit metabolite profile. Front. Plant Sci. 13. doi: 10.3389/fpls.2022.1024588, PMID: 36762178 PMC9907459

[B13] Chen H. Wang Y. Peng Y. Chen G. Wei L. Xu D. . (2025). Pumpkin CmoCDPK20 improved the salt tolerance of grafted cucumber by phosphorylating CmoDREB2A to promote jasmonic acid biosynthesis and antioxidant enzyme activities. Plant J. 122, e70167. doi: 10.1111/tpj.70167, PMID: 40347978

[B14] Cheng G. Chang P. Shen Y. Wu L. El-Sappah A. H. Zhang F. . (2020). Comparing the flavor characteristics of 71 tomato (*Solanum lycopersicum*) accessions in central Shaanxi. Front. Plant Sci. 11, 586834. doi: 10.3389/fpls.2020.586834, PMID: 33362814 PMC7758415

[B10000] Cheng J. Wen S. Xiao S. Lu B. Ma M. Bie Z. (2017). Overexpression of the tonoplast sugar transporter CmTST2 in melon fruit increases sugar accumulation. Journal of Experimental Botany 69 (3), 511–523. 10.1093/jxb/erx440 PMC5853577 29309616

[B17] Gao L. Gonda I. Sun H. Ma Q. Bao K. Tieman D. M. . (2019). The tomato pan-genome uncovers new genes and a rare allele regulating fruit flavor. Nat. Genet. 51, 1044–1051. doi: 10.1038/s41588-019-0410-2, PMID: 31086351

[B18] Gómez-Martínez H. Gil-Muñoz F. Bermejo A. Zuriaga E. Badenes M. L. (2021). Insights of phenolic pathway in fruits: transcriptional and metabolic profiling in apricot (*Prunus Armeniaca*). Int. J. Mol. Sci. 22, 3411. doi: 10.3390/ijms22073411, PMID: 33810284 PMC8037730

[B19] Gonda I. Bar E. Portnoy V. Lev S. Burger J. Schaffer A. A. . (2010). Branched-chain and aromatic amino acid catabolism into aroma volatiles in *Cucumis melo* L. fruit. J. Exp. Bot. 61, 1111–1123. doi: 10.1093/jxb/erp390, PMID: 20065117 PMC2826658

[B20] Guo K. Zhao J. Fang S. Zhang Q. Nie L. Zhao W. (2024). The effects of different rootstocks on aroma components, activities and genes expression of aroma-related enzymes in oriental melon fruit. PeerJ 12, e16704. doi: 10.7717/peerj.16704, PMID: 38192601 PMC10773451

[B22] Huang Y. Li W. Shen T. Chen H. Kong Q. Nawaz M. A. . (2018). Sucrose metabolism in watermelon fruit is affected by fruit-setting method under plastic film greenhouses. J. Hortic. Sci. Biotechnol. 93, 585–594. doi: 10.1080/14620316.2017.1419834, PMID: 37339054

[B21] Huang Y. Li W. Zhao L. Shen T. Sun J. Chen H. . (2017). Melon fruit sugar and amino acid contents are affected by fruit setting method under protected cultivation. Sci. Hortic. (Amsterdam). 214, 288–294. doi: 10.1016/j.scienta.2016.11.055, PMID: 38826717

[B23] Jenkins T. Cowan J. Rivard C. Pliakoni E. (2022). Effect of rootstock on ‘Tasti-Lee’ tomato yield and fruit quality in a high tunnel production system. HortScience 57, 1235–1241. doi: 10.21273/HORTSCI16634-22

[B26] Kaleem M. M. Nawaz M. A. Alam S. M. Ding X. Cheng J. Bie Z. (2023). Rootstock-scion interaction mediated impact on fruit quality attributes of thick-skinned melon during storage under different temperature regimes. Sci. Hortic. (Amsterdam). 312, 111823. doi: 10.1016/j.scienta.2022.111823, PMID: 38826717

[B24] Kaleem M. M. Nawaz M. A. Ding X. Wen S. Shireen F. Cheng J. . (2022). Comparative analysis of pumpkin rootstocks mediated impact on melon sensory fruit quality through integration of non-targeted metabolomics and sensory evaluation. Plant Physiol. Biochem. 192, 320–330. doi: 10.1016/j.plaphy.2022.10.010, PMID: 36302334

[B25] Kaleem M. M. Zhu P. Ateeq M. Li S. Wang J. Cheng J. . (2025). Integrated multi-omics analysis provides molecular insights into flavor variation in melons grafted onto two different pumpkin rootstocks during fruit development. Hortic. Plant J. 11, 1181–1197. doi: 10.1016/j.hpj.2024.02.008, PMID: 38826717

[B27] Khan M. A. Liu D.-H. Alam S. M. Zaman F. Luo Y. Han H. . (2023). Molecular physiology for the increase of soluble sugar accumulation in citrus fruits under drought stress. Plant Physiol. Biochem. 203, 108056. doi: 10.1016/j.plaphy.2023.108056, PMID: 37783072

[B28] Khan M. A. Zaman F. Liu Y.-Z. Alam S. M. Liu D.-H. Han H. . (2025). Citrus NAC47 modulates vacuolar storage of soluble sugars by interacting with type I H^+^-pyrophosphatase genes under drought stress. Physiol. Plant 177, e70303. doi: 10.1111/ppl.70303, PMID: 40462741

[B29] Kundariya H. Yang X. Morton K. Sanchez R. Axtell M. J. Hutton S. F. . (2020). MSH1-induced heritable enhanced growth vigor through grafting is associated with the RdDM pathway in plants. Nat. Commun. 11, 5343. doi: 10.1038/s41467-020-19140-x, PMID: 33093443 PMC7582163

[B30] Kyriacou M. C. Youssef R. Giuseppe C. Rita Z. Dietmar S. (2017). Vegetable grafting: the implications of a growing agronomic imperative for vegetable fruit quality and nutritive value. Front. Plant Sci. 8, 741. doi: 10.3389/fpls.2017.00741, PMID: 28553298 PMC5427113

[B31] Langmead B. Salzberg S. L. (2012). Fast gapped-read alignment with Bowtie 2. Nat. Methods 9, 357–359. doi: 10.1038/nmeth.1923, PMID: 22388286 PMC3322381

[B32] Lecholocholo N. Shoko T. Manhivi V. E. Maboko M. M. Akinola S. A. Sivakumar D. (2022). Influence of different rootstocks on quality and volatile constituents of cantaloupe and honeydew melons (*Cucumis melo.* L) grown in high tunnels. Food Chem. 393, 133388. doi: 10.1016/j.foodchem.2022.133388, PMID: 35671659

[B33] Li C. Zha W. Li W. Wang J. You A. (2023). Advances in the biosynthesis of terpenoids and their ecological functions in plant resistance. Int. J. Mol. Sci. 24, 16. doi: 10.3390/ijms241411561, PMID: 37511319 PMC10380271

[B34] Ling L. Dong T. Liu X. Dong Z. Qiu X. Rong Y. (2019). Effect of nitrogen supply on nitrogen metabolism in the citrus cultivar ‘Huangguogan’. PloS One 14, e0213874. 30897177 10.1371/journal.pone.0213874 PMC6428318

[B35] Liu D. Chen J. Hao Y. Yang X. Chen R. Zhang Y. (2023). Effects of extreme root restriction on the nutritional and flavor quality, and sucrose metabolism of tomato (*Solanum lycopersicum* L.). Horticulturae 9, 813. doi: 10.3390/horticulturae9070813, PMID: 30654563

[B36] Liu X. Sang T. Tang J. Wang Y. Fu Z. Zhang K. . (2025). Combined transcriptomic and metabolomic analysis reveals the regulatory mechanism of pumpkin rootstocks on fruit quality of grafted cucumbers. Scientia Hortic. 347, 114189. doi: 10.1016/j.scienta.2025.114189, PMID: 38826717

[B37] Livak K. J. Schmittgen T. D. (2001). Analysis of relative gene expression data using real-time quantitative PCR and the 2(-Delta Delta C(T)) method. Methods 25, 402–408. doi: 10.1006/meth.2001.1262, PMID: 11846609

[B38] Ma L. Wang Q. Zheng Y. Guo J. Yuan S. Fu A. . (2022). Cucurbitaceae genome evolution, gene function, and molecular breeding. Horticulture Res. 9, uhab057. doi: 10.1093/hr/uhab057, PMID: 35043161 PMC8969062

[B39] Maoz I. Lewinsohn E. Gonda I. (2022). Amino acids metabolism as a source for aroma volatiles biosynthesis. Curr. Opin. Plant Biol. doi: 10.1016/j.pbi.2022.102221, PMID: 35533493

[B40] Miao L. Di Q. Sun T. Li Y. Yu X. (2019). Integrated metabolome and transcriptome analysis provide insights into the effects of grafting on fruit flavor of cucumber with different rootstocks. Int. J. Mol. Sci. 20, 3592. doi: 10.3390/ijms20143592, PMID: 31340498 PMC6678626

[B41] Mindnich R. D. Penning T. M. (2009). Aldo-keto reductase (AKR) superfamily: genomics and annotation. Hum. Genomics. doi: 10.1186/1479-7364-3-4-362, PMID: 19706366 PMC3206293

[B42] Mollavali M. Bornke F. (2022). Characterization of trehalose-6-phosphate synthase and trehalose-6-phosphate phosphatase genes of tomato (*Solanum lycopersicum* L.) and analysis of their differential expression in response to temperature. Int. J. Mol. Sci. 23. doi: 10.3390/ijms231911436, PMID: 36232739 PMC9569751

[B43] Nawaz M. A. Wang L. Jiao Y. Chen C. Zhao L. Mei M. . (2017). Pumpkin rootstock improves nitrogen use efficiency of watermelon scion by enhancing nutrient uptake, cytokinin content, and expression of nitrate reductase genes. Plant Growth Regul. 82, 233–246. doi: 10.1007/s10725-017-0254-7, PMID: 30311153

[B44] Ogunbusola E. M. Alabi O. O. Araoye K. T. Sanni T. A. Jaiyeoba C. N. Adebayo-Alabi I. B. . (2022). Impact of extraction methods on the quality, physicochemical, and functional properties of white melon (*Cucumeropsis mannii*) seed protein concentrates. Food Chem. Adv. 1, 100131. doi: 10.1016/j.focha.2022.100131, PMID: 38826717

[B45] Ouyang B. Wang G. Zhang N. Zuo J. Huang Y. Zhao X. (2023). Recent advances in β-Glucosidase sequence and structure engineering: A brief review. Molecules 28. doi: 10.3390/molecules28134990, PMID: 37446652 PMC10343665

[B47] Sarkar A. K. Sadhukhan S. (2022). Imperative role of trehalose metabolism and trehalose-6-phosphate signalling on salt stress responses in plants. Physiologia Plantarum 174, e13647. doi: 10.1111/ppl.13647, PMID: 35141895

[B48] Shireen F. Nawaz M. A. Xiong M. Ahmad A. Sohail H. Chen Z. . (2020). Pumpkin rootstock improves the growth and development of watermelon by enhancing uptake and transport of boron and regulating the gene expression. Plant Physiol. Biochem. 154, 204–218. doi: 10.1016/j.plaphy.2020.06.003, PMID: 32563044

[B49] Tikunov Y. M. Roohanitaziani R. Meijer-Dekens F. Molthoff J. Paulo J. Finkers R. . (2020). The genetic and functional analysis of flavor in commercial tomato: the FLORAL4 gene underlies a QTL for floral aroma volatiles in tomato fruit. Plant J. 103, 1189–1204. doi: 10.1111/tpj.14795, PMID: 32369642 PMC7496274

[B51] Wan H. Zhang X. Wang P. Qiu H. Guo Y. Cheng Y. . (2022). Integrated multi-omics analysis of developing ‘Newhall’ orange and its glossy mutant provide insights into citrus fragrance formation. Hortic. Plant J. 8, 435–449. doi: 10.1016/j.hpj.2021.12.002, PMID: 38826717

[B50] Wang J. Kaleem M. M. Zhang H. Tian B. Wang Y. Liu B. . (2025). Flavor improvement in melon: A metabolomic study of grafting with wild melon and hybrid pumpkin rootstocks. Hortic. Plant J. 11, 1707–1710. doi: 10.1016/j.hpj.2025.04.002, PMID: 38826717

[B52] Yan Y. Wang W. Hu T. Hu H. Wang J. Wei Q. . (2023). Metabolomic and transcriptomic analyses reveal the effects of grafting on nutritional properties in eggplant. Foods 12, 3082. doi: 10.3390/foods12163082, PMID: 37628081 PMC10453275

[B53] Yang P. M. He S. T. Jiang L. N. Chen X. J. Li Y. F. Zhou J. G. (2020). The effects of pumpkin rootstock on photosynthesis, fruit mass, and sucrose content of different ploidy watermelon (*Citrullus lanatus*). Photosynthetica 58, 1150–1159. doi: 10.32615/ps.2020.068

[B54] Yaschenko A. E. Alonso J. M. Stepanova A. N. (2024). Arabidopsis as a model for translational research. Plant Cell 37, koae065. doi: 10.1093/plcell/koae065, PMID: 38411602 PMC12082644

[B55] Yu J. Sun H. Zhang J. Hou Y. Zhang T. Kang J. . (2020). Analysis of aldo-keto reductase gene family and their responses to salt, drought, and abscisic acid stresses in *Medicago truncatula*. Int. J. Mol. Sci. 21, 754. doi: 10.3390/ijms21030754, PMID: 31979344 PMC7037683

[B56] Zaman F. Liu D.-H. Liu Y.-Z. Khan M. A. Alam S. M. Luo Y. . (2025). Short-day shading increases soluble sugar content in citrus fruit primarily through promoting sucrose distribution, starch degradation and sucrose storage ability. Plant Physiol. Biochem. 223, 109779. doi: 10.1016/j.plaphy.2025.109779, PMID: 40121725

[B58] Zhang H. Li X. Yu H. Zhang Y. Li M. Wang H. . (2019). A high-quality melon genome assembly provides insights into genetic basis of fruit trait improvement. iScience 22, 16–27. doi: 10.1016/j.isci.2019.10.049, PMID: 31739171 PMC6864349

[B57] Zhang H. Liang Q. Chen J. Wang J. Huang Y. Liu B. . (2025). Defense Responses in Prickly Pear (*Cucumis metuliferus*) to *Meloidogyne incognita:* Insights from Transcriptomics and Metabolomics Analysis. Agronomy 15, 1965. doi: 10.3390/agronomy15081965, PMID: 30654563

[B59] Zhang M. Liu W. Wang C. Lin S. Chen Y. Cui H. . (2025). Root-to-shoot mobile mRNA CmoKARI1 promotes JA-Ile biosynthesis to confer chilling tolerance in grafted cucumbers. Nat. Commun. 16, 7782. doi: 10.1038/s41467-025-63228-1, PMID: 40835858 PMC12368190

[B60] Zhong Y. Shi J. Zheng Z. Nawaz M. A. Chen C. Cheng F. . (2019). NMR-based fruit metabonomic analysis of watermelon grafted onto different rootstocks under two potassium levels. Sci. Hortic. (Amsterdam). 258, 108793. doi: 10.1016/j.scienta.2019.108793, PMID: 38826717

[B61] Zhou Y. Li K. Wen S. Yang D. Gao J. Wang Z. . (2023). Phloem unloading in cultivated melon fruits follows an apoplasmic pathway during enlargement and ripening. Horticulture Res. 10. doi: 10.1093/hr/uhad123, PMID: 37554344 PMC10405131

